# Leprosy in the Democratic Republic of the Congo: a persistent but neglected disease – current trends, challenges and future prospects

**DOI:** 10.1097/MS9.0000000000003540

**Published:** 2025-07-02

**Authors:** Christian Tague, Hermann Yokolo, Joshua Ekouo, Dujardin Makeda, Nathan Mugenyi, Shahar Bano, Gbènonminvo Enoch Cakpo, Farheen Naaz, Alimasi Kashafali, Elie Kihanduka, Aymar Akilimali

**Affiliations:** a Department of Research, Medical Research Circle (MedReC), Goma, DR Congo; b Faculty of Medicine, Mbarara University of Science and Technology, Mbarara, Uganda; c Department of Pharmacy, Faculty of Health Sciences, Victoria University, Kampala, Uganda; d Department of Medicine, Islamic International medical college, Pakistan; e Higher Institute of Population Sciences, Joseph KI-ZERBO University, Ouagadougou, Burkina Faso; f Medical College, Deccan College of Medical Sciences, Hyderabad, India; g International Veterinary Vaccinology Network, The Roslin Institute University of Edinburgh, Edinburgh, United Kingdom; h Global Schistosomiasis Alliance, London, United Kingdom

Leprosy, although often perceived as a historical disease, remains a pressing public health issue in the Democratic Republic of the Congo (DR Congo). Despite decades of global eradication efforts, this chronic infectious disease caused by *Mycobacterium leprae (M. Leprae)* continues to affect thousands, particularly in impoverished and remote communities. According to the World Health Organization (WHO), the DR Congo reported 2123 new leprosy cases in 2021^[1]^, and 2045 cases in 2022, with a detection rate of 2.4 per 100 000 population. While global prevalence has declined, incidence rates in DR Congo have remained stagnant over the past decade, particularly in provinces such as Kwilu, Tshopo, Kasaï, Équateur, and Haut-Katanga. These areas consistently report case detection rates above the national average, indicating persistent transmission in focal hotspots. Such patterns underscore the need for disaggregated epidemiological data to guide interventions, yet data collection remains inconsistent.

The persistence of leprosy in the DR Congo is driven by multiple, interrelated factors. Socioeconomic determinants such as extreme poverty, overcrowding, poor sanitation, and malnutrition play a central role in sustaining transmission^[2]^. In many endemic areas, people live in conditions that promote close contact and reduce immunity, increasing both exposure and susceptibility to *M. leprae*. Households in these zones often survive on less than $1.90 per day, making access to transportation or healthcare facilities unaffordable. Malnutrition especially among children and women further compromises immune responses, heightening vulnerability to infection and complicating recovery^[3]^. Moreover, forced displacement, conflict, and rural isolation limit the reach of public health interventions and further entrench poverty-related risks.

Despite efforts, health system barriers severely impede effective leprosy control. A significant proportion of rural health zones lack the necessary capacity for early diagnosis, diseases surveillance, or long-term follow-up^[4]^. Chronic underfunding of the health sector estimated at less than 4% of GDP alongside a shortage of trained personnel and diagnostic tools, limits early detection and comprehensive care. Only one-third of health facilities in endemic provinces are equipped to detect leprosy cases accurately. Referral pathways are fragmented, and stockouts of multidrug therapy (MDT) are not uncommon. Compared to countries like Nepal or Brazil, which have successfully reduced leprosy prevalence through decentralized, integrated, and community-based strategies^[5]^, the DR Congo lacks a unified and adequately resourced national approach to leprosy elimination. Health information systems in DR Congo remain paper-based in most rural settings, further constraining data-driven planning.

Stigma and social discrimination remain entrenched barriers to timely diagnosis and treatment. Affected individuals often face exclusion from family and community life, job loss, school dropout, and even violence^[6]^. These experiences lead to delays in seeking care or outright refusal of treatment. The psychosocial burden of leprosy is substantial and largely neglected in existing program responses^[7]^. A qualitative study in Kasaï province documented widespread misconceptions about leprosy as divine punishment, prompting isolation of patients even after treatment. In contrast, community-based stigma reduction initiatives in India and Brazil such as peer support groups, patient advocacy networks, and school-based education have shown that empowering affected individuals and promoting inclusive messaging can enhance health-seeking behaviors and reduce community-level discrimination^[8]^. The DRC could benefit from piloting such models in endemic zones, particularly by partnering with local religious leaders and traditional healers to dispel myths.

While the Ministry of Health, WHO, and several NGOs have supported programs for active case detection, MDT distribution, and community sensitization^[9]^, many of these interventions remain fragmented, donor-dependent, and unsustainable. Health worker training is sporadic, outreach efforts are often limited in scope, and the existing surveillance systems are inadequate for monitoring disease trends at the sub-national level^[10]^. Furthermore, program data are not consistently disaggregated by age, sex, or geography, limiting the ability to target high-risk populations or measure program impact effectively. A lack of electronic reporting tools delays feedback loops and compromises planning.

To address these challenges and advance toward leprosy elimination, a comprehensive and multisectoral strategy is required. First, epidemiological surveillance must be strengthened through improved data collection, integration of digital health platforms, and regular publication of sub-national trends^[11]^. Sentinel surveillance sites in high-burden provinces should be established to track incidence trends and antimicrobial resistance. Second, leprosy services must be integrated into the primary healthcare system to ensure consistent access to diagnosis, treatment, and rehabilitation. Every health facility in endemic zones should be staffed with trained personnel and equipped with necessary supplies, including MDT and diagnostic kits^[12]^. Third, national budget allocation for neglected tropical diseases, including leprosy, must be increased to reduce reliance on international donors and ensure sustainability^[13]^. Fourth, anti-stigma campaigns should be launched through schools, churches, mosques, and local radio stations, emphasizing the curability of leprosy and promoting narratives of recovery. These efforts should include training for community health workers on psychosocial support and patient rights. Fifth, successful strategies from other endemic countries such as community-based care, contact tracing, and decentralization should be adapted to the DR Congo context^[14,15]^. Sixth, collaboration between government, NGOs, and international health agencies must be formalized through multisectoral task forces to ensure coordination and accountability. Finally, each recommendation should be accompanied by an operational framework defining short-, medium-, and long-term objectives. For example, the expansion of diagnostic capacity in endemic areas could be planned over a 3-year period, with annual targets for staff training, distribution of diagnostic kits, and integration into the primary health system. A dedicated budget, estimated at approximately USD 1 to 2 million per year for priority provinces (based on expenditures observed in comparable programs in Nepal and Brazil), would ensure the sustainability of interventions. An integrated monitoring and evaluation mechanism, based on indicators of coverage, detection time, and social reintegration, is also essential to adjust strategies in real time.

In conclusion, leprosy continues to be a persistent and neglected disease in the DR Congo, disproportionately affecting vulnerable populations in rural and underserved regions. While some progress has been made particularly through the availability of free treatment and awareness campaigns numerous obstacles, including poverty, limited health infrastructure, and entrenched stigma, continue to drive transmission and delay recovery. The situation demands urgent and coordinated action. The government must lead a revitalized national strategy, with clearly defined targets, adequate funding, and stakeholder engagement. International partners must support not only with resources but also technical expertise and best practices. A data-driven, equity-focused, and integrated approach combining early detection, effective treatment, stigma reduction, and systemic health system strengthening is essential. With political will and collaborative partnerships, leprosy elimination in the DR Congo is an achievable goal.

## Data Availability

Not applicable.
